# Immune Myocarditis Overlapping With Myasthenia Gravis Due to Anti-PD-1 Treatment for a Chordoma Patient: A Case Report and Literature Review

**DOI:** 10.3389/fimmu.2021.682262

**Published:** 2021-07-08

**Authors:** Shujing Liang, Jingxian Yang, Yun Lin, Tong Li, Wenrong Zhao, Jun Zhao, Chunyan Dong

**Affiliations:** ^1^ Department of Oncology, Shanghai East Hospital, School of Medicine, Tongji University, Shanghai, China; ^2^ Department of Nuclear Medicine, Shanghai East Hospital, School of Medicine, Tongji University, Shanghai, China

**Keywords:** chordoma, immune checkpoint inhibitor, PD-1 blockade, sintilimab, myocarditis, myasthenia gravis

## Abstract

Immunotherapy begins to be widely used due to the increasing exploration and gratifying effects in multiple cancers. Chordoma, as a rare bone malignant tumor, often recurs and metastasizes after undergoing surgery and radiotherapy. Therefore, immunotherapy can be explored as an emerging, potentially effective treatment to improve the survival rate and clinical benefit of patients. However, a variety of immune-related adverse events (irAEs) cannot be avoided completely. And the immunotherapy-induced myocarditis, as a rare but fatal irAE, has been increasingly reported. Understanding the mechanism involved in irAEs can inform best practices for side effects management. Here, we firstly reported a case of immune myocarditis and subsequent myasthenia gravis (MG) following anti-PD-1 treatment for chordoma.

## Introduction

Chordoma, a rare primary malignant bone tumor, is often found in skull base and axial skeleton (1, 2). It is considered to originate from notochord remnants and behave as local destructive invasion though mainly with low-grade malignancy (1, 3). Its classic treatments are known as surgical resection and radiotherapy (1). But due to the high recurrence rate, high metastasis rate after recurrence, low survival rate and the complexity of this rare disease, there is no approved treatment consensus or systematic therapies (2–4). Thus, emerging therapies, such as targeted therapy and immunotherapy, have entered the field in order to bring more clinical benefits for patients.

Immunotherapy, especially following by the clinical success of immune checkpoint blockade therapy, has been regarded as a potentially effective anti-tumor therapy. Anti–programmed cell death-1/ligand-1 (PD-1/PD-L1) and anti–cytotoxic T lymphocyte antigen-4 (CTLA-4) monoclonal antibodies (mAbs) have been approved by U.S. Food and Drug Administration (FDA), having a wide range of applications. Several researches have explored the expression of PD-1/PD-L1 in chordoma recently, indicating that the combined use of immune checkpoint inhibitors (ICIs) may be a more effective treatment. However, immune-related adverse events (irAEs) could occur (5). Myocarditis caused by ICIs is a rare but fatal irAE that cannot be ignored during the whole course of disease management (6, 7).

Here, we report a case of ICI-induced myocarditis after the treatment of anti-PD-1 therapy for chordoma. A 77-year-old gentleman with relapsed chordoma mainly complained about an acute chest tightness, dyspnea and subsequent ptosis three weeks after the use of sintilimab. After completing necessary examinations and excluding possible causes one by one, he was diagnosed as ICI-induced myocarditis overlapping with MG. Then, his symptoms were resolved after an immunosuppressive therapy with high-dose steroids. Our report aims to raise awareness of the early prediction, early intervention and correct treatments in ICIs-induced rare side effects during the immune checkpoint blockade treatments of rare tumor. It also adds information to the database of such rare irAEs to facilitate further research and exploration.

## Case Presentation

**Figure 1:**
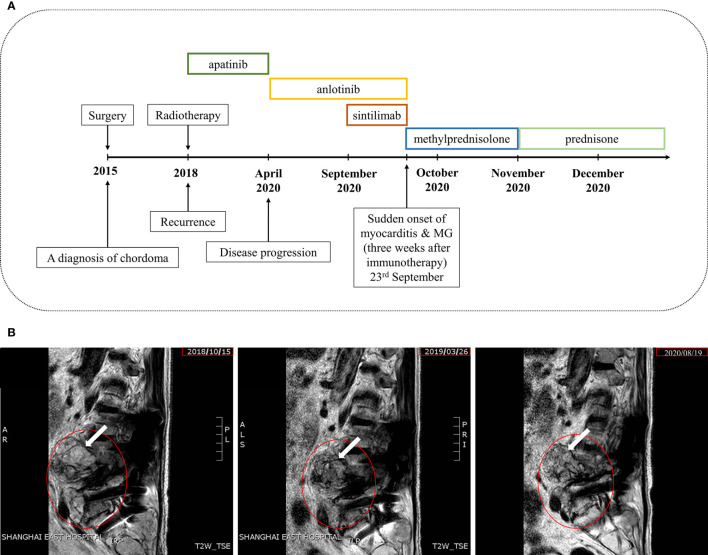
Case presentation. **(A)** Timeline of disease diagnosis and treatment. **(B)** Magnetic resonance imaging (MRI) from 2018 to 2020 (from left to right) in T2WI. The red circle indicates the damaged lesion and the white arrow indicates the tumor site.

A 77-year-old male with relapsed chordoma suffered from an acute onset of dyspnea and then was presented to the emergency department in the hospital. Five years earlier, the chordoma was diagnosed and treated surgically. In June 2018, a recurrence of chordoma was diagnosed *via* the puncture pathology guided by ultrasound. During this period, the patient experienced soreness and numbness in the left lower limb after walking about 500 meters long without obvious causes, from the back of the thigh to the heel, which could be relieved after rest. Therefore, he paid little attention to this symptom. When he came to hospital for follow-up treatment, he could no longer walk. Then he received intensity-modulated radiation therapy (IMRT) for local recurrence lesions, 200Gy/f daily, and the total dose of 4000Gy/20f. After that, he started regular maintenance treatment with apatinib (250mg/d) by oral administration. Until April and August 2020, disease progressions were detected twice. Apatinib was replaced with anlotinib for targeted therapy in April 2020. And in September 2020, the patient was given a combined therapy of sintilimab, a domestic anti-PD-1 mAb, and anlotinib. Three weeks after the use of sintilimab, the patient developed acute chest tightness, shortness of breath, and sweating profusely. Droopy eyelids on both sides presented in the following 5 days. The patient has not experienced irAEs before. An overview of the course of the disease can refer to **Figure 1**.

**Figure 2:**
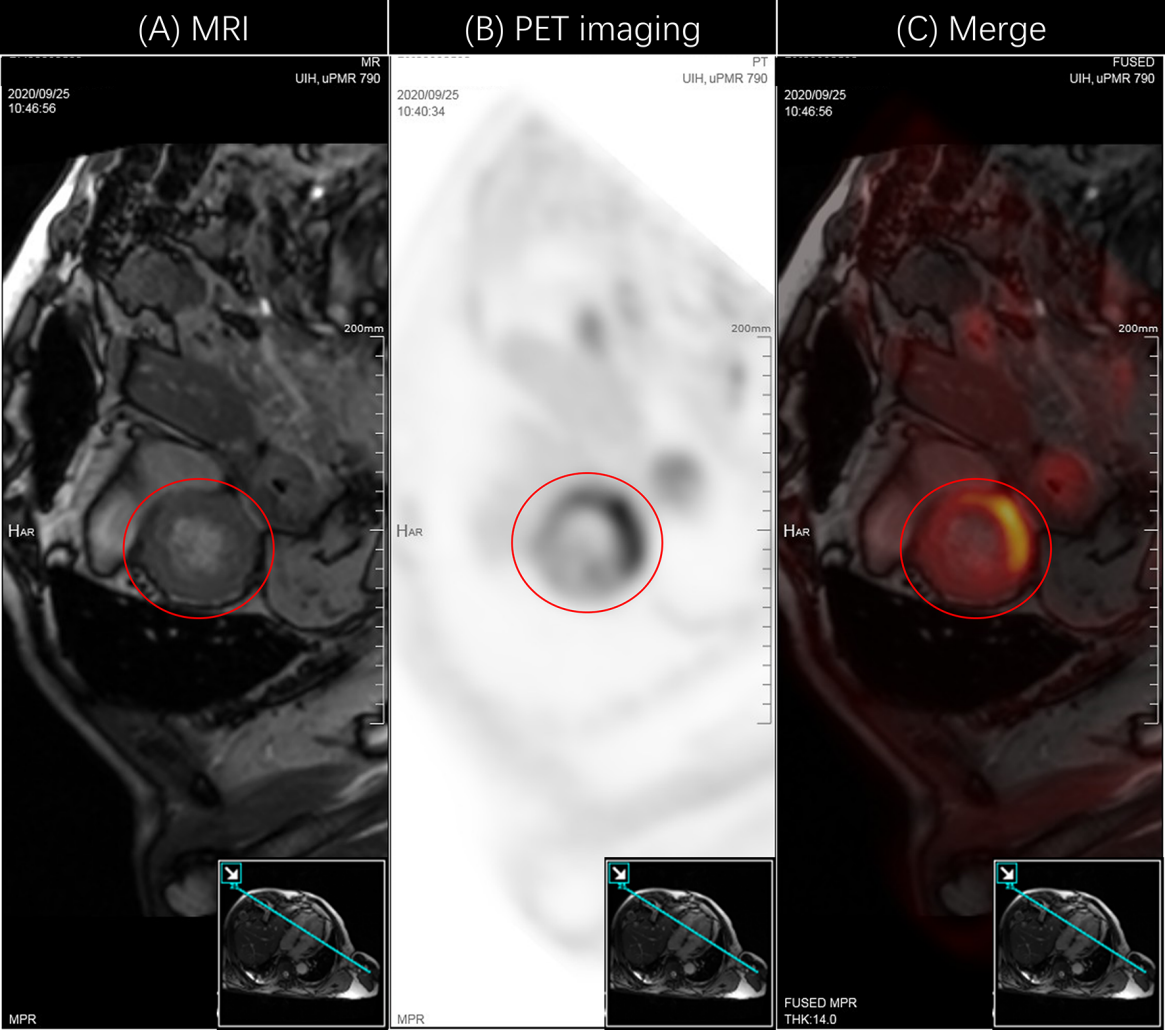
PET-CMR images showed myocardial inflammatory damage. **(A)** MR image indicated the physiological tissue structure of the heart. The red circle represented the left ventricle. The figure in the lower right corner was the image cut surface. **(B)** PET image indicated the uptake of the contrast media ^18^F-FDG. The red circle represented the increased intake. **(C)** Fusion image of PET and MR, which visually displayed myocardial inflammatory damage in the left ventricle. PET, positron emission computed tomography; ^18^F-FDG, ^18^F-flurodeoxyglucose.

**Figure 3:**
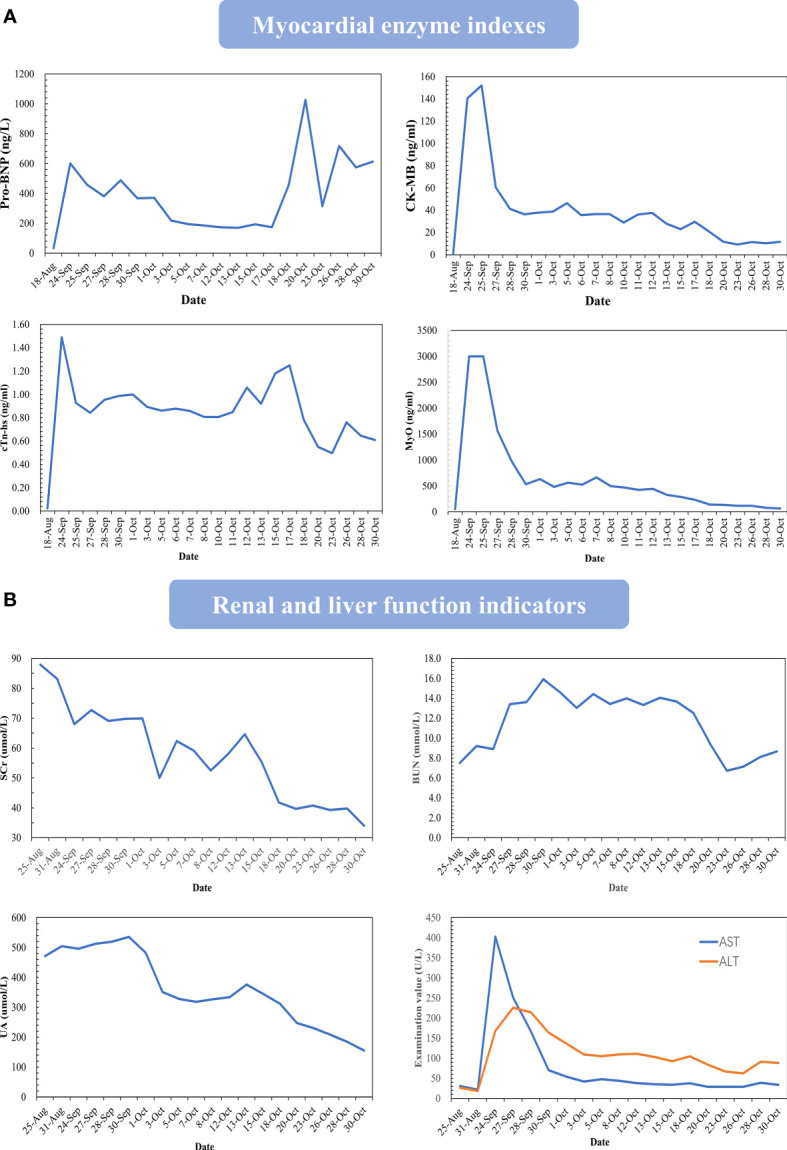
Changes in important indicators during the course of the disease. Changes in myocardial enzymes **(A)**, renal function and liver function **(B)**. Take the value of the routine examination before the onset as the base.

In response to the patient’s condition, we immediately carried out the relevant laboratory examinations, electrocardiogram (ECG) and necessary imaging tests. Myocardial enzyme results suggested that serum brain natriuretic peptide (BNP) 581.8ng/L, creatine kinase-MB form (CK-MB) 140.7ng/ml, troponin T (TnT) 1.29ng/ml, myoglobin (MYO)>3000ng/ml. Initial ECG showed sinus rhythm, ST segment abnormalities (I, II, aVF, V4, V5, V6 upslope and horizontal depression 0.5-1.0mm), T wave changes (aVL inversion) and Q wave in Lead III. Cardiac ultrasonography demonstrated that systolic function of left ventricular (LV) was normal and diastolic function of LV decreased, with a left ventricular ejection fraction (LVEF) of 61%. Positron emission computed tomography-cardiovascular magnetic resonance imaging (PET-CMR) demonstrated myocardial inflammatory and traumatic changes (**Figure 2**). Acute myocardial infarction, which was initially suspected, was excluded. Viral myocarditis and autoimmune diseases were also ruled out through further etiology and autoantibody testing. We next reviewed the medical history and invited Department of Cardiology, Ophthalmology, and Neurology for a multi-disciplinary subject consultation. ICI-mediated myocarditis and MG was highly considered. Therefore, we proposed that endocardial myocardial biopsy (EMB), electromyogram (EMG) and MG related antibodies need to be completed. Only the serological tests were accepted. The testing result indicated that the antibodies against acetylcholine receptor (AChR) and Titin were positive; the antibodies against muscle specific tyrosine kinase (MuSK), voltage-gated calcium channel (VGCC) and low-density lipoprotein receptor-related protein 4 (LRP4) were negative. Based on the above information, immunosuppressive therapy with corticosteroids was given to the patient according to the changes in myocardial enzymes and symptoms (methylprednisolone ivvp. 160mg q8h for 5 days, 80mg q8h for 3 days, 40mg q8h for 7 days, 40mg q12h for 7 days, 40mg qd for 9 days, prednisone p.o 50mg qd). At the same time, symptomatic and supportive treatments such as myocardium, liver, stomach protection, and pain relief were initiated. Important indicators were regularly reviewed throughout the following treatment process (**Figure 3**). Gradually, the patient’s vital signs were relatively stable and he was discharged. After discharge, he received oral prednisone maintenance treatment and regular follow-up as an outpatient. The myocardial enzyme indexes were declining in a fluctuating manner and returned to the normal range after 2 months of follow-up.

From the perspective of the patient, having a near-death experience was very frightening. He felt greatly relieved after 3 days of corticosteroid treatment, and built a relationship with his care team, both of which contributed to lowering his anxiety. The reduced length of intensive care unit (ICU) stay also helped mitigate the financial burden.

## Discussion

Following by the FDA approval of certain ICIs, therapeutic cancer vaccine and chimeric antigen receptor (CAR)-T cell therapy, the 2018 Nobel Prize in Physiology or Medicine implied that immunotherapy for cancer is regarded as a potentially potent therapy in the next decades (8). Anti-PD-1/PD-L1 mAbs are currently the most explored and marketed immunotherapy agents. They have shown promising activity in the application of multiple cancers. With the continuous increase in the number of clinical trials and translation, interaction mode, treatment management, experimental design and irAEs need further consideration (9). Generally acknowledged that, T cell activation requires not only the antigen stimulation but also the synergy of costimulatory factors. PD-1, a member of immunoglobulin (Ig) superfamily, is one of the important negative costimulatory factors with wider expression than other T cell-restricted CD28 family members (10, 11). Its ligands also display different patterns of expression: PD-L1 is expressed on multiple types lymphohematopoietic cells, certain nonhematopoietic cells and nonlymphoid organs such as heart and muscle (12, 13), while PD-L2 is induced by cytokines only on macrophages and dendritic cells (DCs) (10). The non-overlapping characteristics of the two ligands make the different therapeutic roles in regulating immune response and autoimmunity. In the process of exerting respective therapeutic effects, the side effects of anti-PD-1/PD-L pathway are mostly reported as dermatologic toxicity, gastrointestinal toxicity, endocrinopathies, pneumonitis and rheumatologic toxicity (14). Rare irAEs like neurologic toxicity, renal toxicity, ocular toxicity, cardiovascular toxicity and hematologic toxicity have been increasingly reported in recent year (14, 15).

The case reported here is an elderly male patient with recurrence of the rare chordoma. In the absence of better treatment options and the repeated disease progression, the patient and his family members urgently required immunotherapy, although chordoma was contraindicated. Neither personal history nor family history had a clear history of immune-related diseases. He was admitted to the hospital because of an acute onset of dyspnea and the subsequent drooping eyelids. Experimental data showed significantly elevated myocardial enzyme indicators. There were no typical signs indicating acute coronary syndrome in ECG but ST-T segment dynamic changes and no systolic dysfunction of LV in cardiac ultrasonography with a 61% LVEF. Subsequent PET-CMR indicated the myocardial inflammatory damage.

To confirm the diagnosis, we first excluded those fatal emergencies such as acute myocardial infarction, pulmonary embolism and aortic dissection. Especially, the elderly man with high blood pressure should be concerned about acute chest tightness and sweating profusely which can be caused by acute coronary syndrome. Acute pulmonary embolism was often accompanied by sudden chest pain and shortness of breath. The blood D-dimer increased significantly, the partial pressure of carbon dioxide and oxygen in artery (PaCO_2_ and PaO_2_) decreased and the ECG usually indicated right ventricular (RV) injury. Aortic dissection typically presented as tear-like pain in the chest and back which peaked at the beginning, and the location of the pain varied with the extent of the dissection. The patient here had no lasting precordial pain without radiating to the abdomen or lower extremities and it could be self-relieved. The blood pressure of both extremities was symmetrical. The experimental examinations and imaging results did not support the description of these emergencies. Other differential diagnosis of myocarditis includes heart failure, viral myocarditis and cardiomyopathy. The patient mainly complained about recurrent chest tightness and shortness of breath without cough, sputum or oliguria. And the cardiac ultrasound result revealed a slight enlargement of the left atrium and decreased ventricular diastolic function. With intermittent increase in BNP, it should be considered that the patient might have heart failure with normal ejection fraction. Due to the lack of incentives for infection, the negative result found in virus detection and atypical performances in ECG and cardiac ultrasound, viral myocarditis has been temporarily excluded. Since then, we reviewed the medical history and thought that the anti-PD-1 treatment 3 weeks ago might be the cause. Combining the above symptoms, signs, most important PET-CMR findings and multidisciplinary consultation, ICI-induced myocarditis could be diagnosed. For the subsequent ptosis, the diseases that cause oculomotor nerve palsy and blepharospasm should be distinguished. Its common complication known as thymoma also need to be checked. The paralysis in both lower limbs was mainly considered as a result of recurrent chordoma rather than Guillain-Barré syndrome, a typical demyelinating disease. The history of ICI usage, diagnosis of ICI-induced myocarditis and the subsequent positive results of MG-related antibodies indicated the high probability of ICI-induced MG. The patient was then given immunosuppressive therapy with adequate steroids while monitoring myocardial and immune parameters.

Getting insight into the mechanism of the irAEs occurrence can benefit the management strategy in clinical practice. There are two main probable pathogenesis of the ICI-induced myocarditis occurrence. The same antigenic determinants shared between tumor and striated muscle is the first one. Johnson et al. performed an autopsy on two patients with myocarditis which accompanied by myositis after combination immunotherapy (16). Lymphocyte infiltration in tumor site and metastases increased compared to the biopsies before treatment. The same immune infiltration was only observed in myocardium and skeletal muscle without other tissues (16, 17). Through further T-cell receptor next generation sequencing, two patients possessed high-frequency T-cell receptor sequences between striated muscle and tumor infiltrates. Irina et al. observed the same diffuse lymphohistiocytic myocarditis in 6 patients by EMB. The infiltrates were characterized by a population of PD-1–positive, CD8-positive, granzyme-B–positive T lymphocytes (18). They further found the immunoreactivity for PD-L1 in damaged area and proposed that the expression of PD-L1 on myocytes might be the key factor for avoiding self-targeting immune responses, which was in line with the second probable mechanism. That is, the absence or additional blockade of the PD-L1–PD-1 axis breaks peripheral immune tolerance resulting in heart-specific autoimmune-related myocarditis. Multiple research groups carried out experiments on immune checkpoint gene knockout mice (19–21). All affected PD-1^-/-^ mice showed diffuse deposition of immunoglobulin G (IgG), notably IgG1, in heart but no other organs expressing PD-1. The high-titer IgG autoantibodies could react to a specific 33-kD protein extracted from the normal heart under the situation that the mice had normal T/B lymphocyte function. Whether the 33-kD autoantigen acted on transverse (T) tube need further exploration (19). Genetic background and environmental factors also need to be considered (20). For in-depth study, Mariella et al. established a troponin I-directed autoimmune myocarditis mouse model to explore the role of subcellular localized immunoproteasome (including low-molecular-weight protein 2 (LMP2), low-molecular-weight protein 7 (LMP7), and multicatalytic endopeptidase complex subunit 1 (MECL1)) (21). The experimental results revealed that mediating TLR (Toll-like receptor)–MyD88 (myeloid differentiation primary response 88) pathways, dendritic cells (DCs) were stimulated to present self-antigens and monocytes were promoted to secrete proinflammatory cytokines, thereby driving CD4-positive T cells differentiation toward helper T cell 17 (Th17) (interleukin-17) and Th1 (interferon-γ) effector cells (21). They not only stimulated autoantibody production but also reduced regulatory T cells (Treg) pool. In general, ICIs therapy enables the increasing release of tumor-specific T cells, but it also weakens the signal that regulates immune tolerance, leading to the activation of self-reactive effector cells and the heart tissue damage. Especially, the combination immunotherapy with a greater likelihood of fatal outcome caused by immune myocarditis requires more attention.

Clinically, Bonaca et al. are trying to establish a uniform definition of cancer immunotherapies associated myocarditis: on the basis of excluding acute cardiovascular events (e.g., acute coronary syndrome, trauma, etc.) and the standard definition of myocarditis, immune-related myocarditis occurs soon after ICIs treatment and is then classified as definite/probable/possible myocarditis according to pathology, imaging, ECG, syndrome and biomarkers (22). As a result, it can help establish a baseline and follow-up evaluation criteria to identify early or subclinical events in oncology clinical trials. The consistency of collected reports and data is also guaranteed (22). Refer to reference (23, 24) for more diagnostic information. In terms of treatment, except for common respiratory and hemodynamic support treatment, immunosuppressive therapy is the main method dealing with drug-mediated toxicity (25). Especially for grade 3~4 adverse effects, prednisone 1-2 mg/kg/day or equivalent dose treatment should be initiated as soon as possible. Zhang et al. concluded that high initial dose (intravenous methylprednisolone 1000 mg/d) and earlier initiation of corticosteroids (≤ 24 h) were associated with improved cardiac prognosis in ICI-related myocarditis through a retrospective study of 126 clinical cases (26). If the patient’s physical condition continues to deteriorate, immunoglobulin and plasma exchange are available as options. If the patient does not respond well to hormone shock, other immunosuppressive treatments such as azathioprine, tacrolimus, mycophenolate, methotrexate or cytokine antagonists need to be used as combined therapy. Rituximab, infliximab and tocilizumab have been reported as potentially effective drugs for severe steroid resistant ICI-related myocarditis in combined therapy (7, 17, 25). Simultaneously, stop immune treatment immediately until the symptoms are relieved. Doctors should reassess the patient’s overall situation to decide whether to continue immunotherapy. Cardiac magnetic resonance and late gadolinium enhancement (LGE) can help evaluate the prognosis of acute myocarditis in patients with preserved LVEF (27, 28). It is worth noting that more recent data indicate the reduced cancer survival after the high-dose corticosteroids treatment, which is different from the initial research data (26). Therefore, it is important to continuously explore the impact of high-dose corticosteroids on the outcome of cancer patients who are treated with ICIs. The development of subunit targeted therapy for ICI-induced myocarditis and the prevention of such irAEs during the whole treatment period have become new research directions.

For the treatment of the primary disease and accompanied myositis, a brief introduction is given below. Chordoma, a locally advanced disease, is treated on the basis of surgical resection and radiotherapy (1). When it recurs locoregionally, systemic treatment can be selected (3). Cytotoxic chemotherapy is usually ineffective (29). The effect of inhibitors of potentially relevant therapeutic targets such as mammalian target of rapamycin (mTOR), β-type platelet-derived growth factor receptor (PDGFRB), epidermal growth factor receptor (EGFR), and MET (30), has been exploring in laboratory and clinical trials (3, 29). Currently, it suggested that imatinib and combined therapy with imatinib may have some anti-chordoma activity. An effective strategy is critical for chordoma and the burgeoning immunotherapy including tumor antigen vaccines and immune checkpoint blockade may also create new treatment paradigms because of its interaction mode and clinical success (31). The treatments of MG can mainly be divided into two categories: symptomatic drug treatment (like pyridostigmine, neostigmine, ambenonium chloride etc.) and immunosuppressive drug treatment (like corticosteroids, immunoglobulin exchange, plasma exchange, immunosuppressive mAbs etc.) (32). But in the presented case, immunosuppressive therapy for the essential cause that led to MG is the most important. We reflected that the performance of neostigmine test and symptomatic treatment before the accurate diagnosis of MG may bring a better outcome. The different types of MG-related antibodies can also guide the option of treatment plans (33).

In conclusion, ICIs therapy can induce a spectrum of immune side effects including the rare but fatal myocarditis. Although mechanism of ICIs-related cardiotoxicity has not been fully understood and the consensus on diagnosis and treatment remains to be reached, doctors treating patients with ICIs should be wary of potentially fatal cardiotoxic effects, especially due to its characteristic of early onset, non-specific symptoms, and fulminant progression. The history of heart disease or autoimmune disease should not be ignored. Timely and appropriate preventive and therapeutic treatments is essential to avoid fatal outcomes. The accumulation of each rare case provides useful pathogenesis and clinical experience in patient management that have no valuable strategies and guidelines to rely on.

## Data Availability Statement

The raw data supporting the conclusions of this article will be made available by the authors, without undue reservation.

## Ethics Statement

Written informed consent was obtained from the individual(s) for the publication of any potentially identifiable images or data included in this article.

## Author Contributions

SL and JY contributed equally to this work. SL provided case information and contributed to data analysis and manuscript revision. JY drafted the manuscript. TL, WZ, YL, and JZ performed the clinical management of the patient. CD contributed to the project development. All authors contributed to the article and approved the submitted version.

## Funding

This work was supported by the National Natural Science Foundation of China (81860547, 82073387, 81703075).

## Conflict of Interest

The authors declare that the research was conducted in the absence of any commercial or financial relationships that could be construed as a potential conflict of interest.
